# Lyso-Sulfatide Binds Factor Xa and Inhibits Thrombin Generation by the Prothrombinase Complex

**DOI:** 10.1371/journal.pone.0135025

**Published:** 2015-08-11

**Authors:** Subramanian Yegneswaran, Yajnavalka Banerjee, José A. Fernández, Hiroshi Deguchi, John H. Griffin

**Affiliations:** Department of Molecular and Experimental Medicine, The Scripps Research Institute, La Jolla, California, United States of America; University of Pennsylvania School of Medicine, UNITED STATES

## Abstract

Blood coagulation reactions are strongly influenced by phospholipids, but little is known about the influence of sphingolipids on coagulation mechanisms. Lysosulfatide (lyso-SF) (sulfogalactosyl sphingosine) prolonged factor Xa (fXa) 1-stage plasma clotting assays, showing it had robust anticoagulant activity. In studies using purified clotting factors, lyso-SF inhibited >90% of prothrombin (II) activation for reaction mixtures containing fXa/factor Va (fVa)/II, and also inhibited II activation generation by fXa/ phospholipids and by Gla-domainless-fXa/fVa/phospholipids. When lyso-SF analogs were tested, results showed that N-acetyl-sulfatide was not anticoagulant, implying that the free amine group was essential for the anticoagulant effects of lyso-SF. Lyso-SF did not inhibit fXa enzymatic hydrolysis of small peptide substrates, showing it did not directly inhibit the fXa activity. In surface plasmon resonance studies, lyso-SF bound to immobilized inactivated fXa as well as inactivated Gla-domainless-fXa. Confirming this lyso-SF:fXa interaction, fluorescence studies showed that fluorescently-labeled-fXa in solution bound to lyso-SF. Thus, lyso-SF is an anticoagulant lipid that inhibits fXa when this enzyme is bound to either phospholipids or to fVa. Mechanisms for inhibition of procoagulant activity are likely to involve lyso-SF binding to fXa domain(s) that are distinct from the fXa Gla domain. This suggests that certain sphingolipids, including lyso-SF and some of its analogs, may down-regulate fXa activity without inhibiting the enzyme’s active site or binding to the fXa Gla domain.

## Introduction

Prothrombin (II) is cleaved at two Arg residues, namely at position 271 and 320, by the enzyme factor Xa (fXa) of the prothrombinase complex (II-ase, comprising fXa factor Va phospholipids(PL)), in the penultimate step of blood coagulation [1,2]. The product of this reaction, thrombin (IIa), is a serine protease that is essential for clot formation. Factor Va (fVa) serves as a cofactor in this reaction and accelerates the production of IIa by the II-ase complex by five orders of magnitude [1,3,4]. FXa is strategically positioned at the junction of the intrinsic and extrinsic pathways and proximal to IIa in the coagulation cascade such that targeting fXa with new drugs can have profound effects on coagulation and venous thromboembolism risk [5–8]. Although the new oral anticoagulants are beneficial, serious bleeding tendencies, especially at higher doses of fXa inhibitors, have been reported and some argue that lab-based dosing of fXa inhibitors would reduce serious bleeding [9]. Thus, additional knowledge about regulation of fXa may have direct clinical relevance.

Membrane phospholipid (PL) surfaces were long thought to play only a passive role in the II-ase reaction by providing a surface template for the assembly of the enzyme•cofactor•substrate multiprotein complex [1,2]. Variations in PL composition modulate fXa activity [10,11], and calcium ions dynamically affect PL conformation and promote binding to clotting factors to membrane bilayers [12,13]. However, functionally important conformational changes are allosterically induced in fXa by its binding to either PL or to fVa [14,15]. Furthermore, short-chain phosphatidylserine (PS), which is essentially soluble, promotes fXa activity and binds to domains in fXa other than the PL-binding Gla domain [16,17], indicating that allosteric conformational changes which increase activity in fXa may be induced by lipid binding to sites outside the protein’s Gla domain.

**Fig 1:**
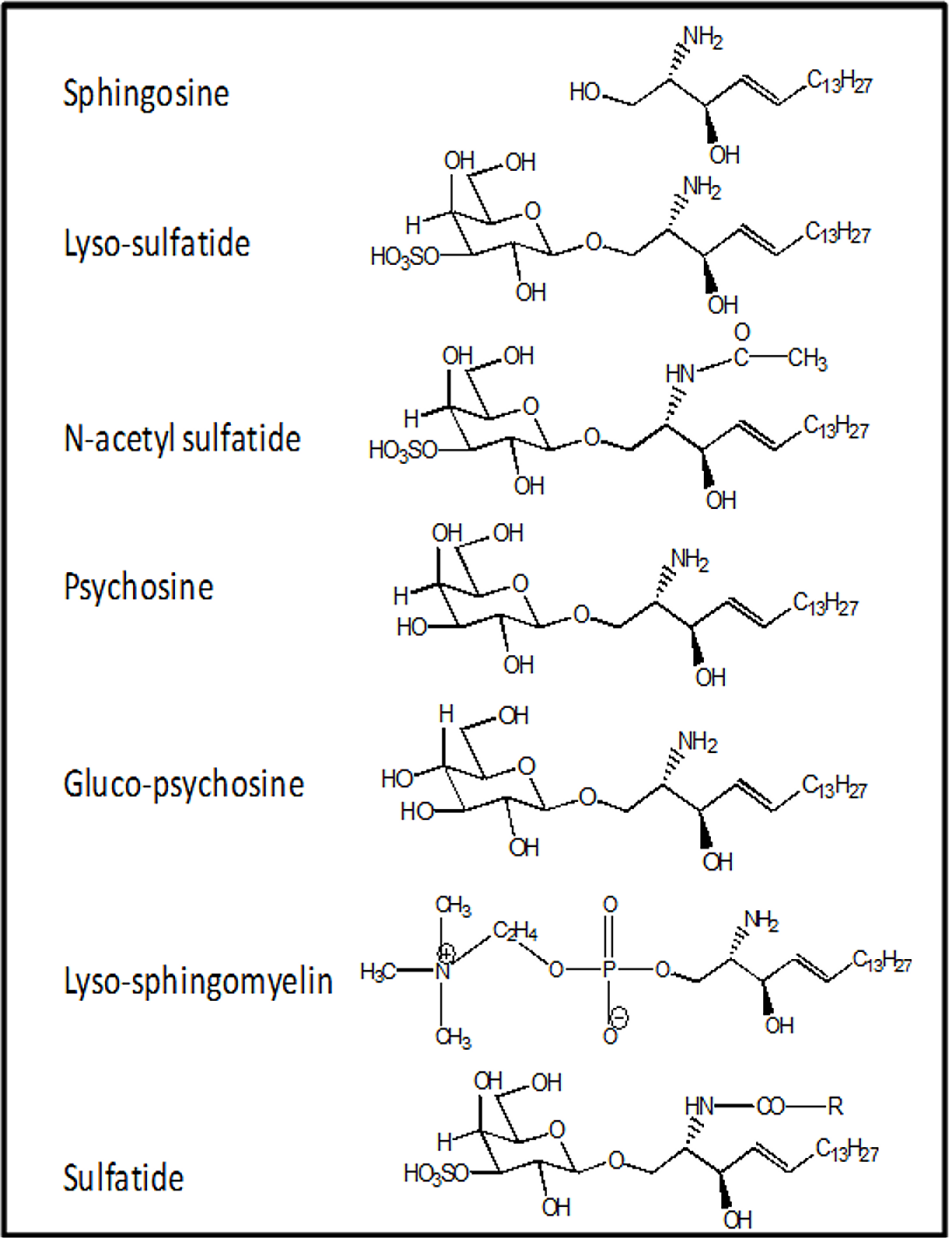
Structures of Lyso-SF and Its Analogs.

Sphingolipids and glycosphingolipids are essential components of blood cells and plasma as well as every cell type, and their levels are determined by highly dynamic metabolic pathways which are subjects of much ongoing research [18]. Sphingolipids and their metabolism are intimately involved in apoptosis and autophagy [19]. Knowledge about potential influences of sphingolipids on blood coagulation reactions is very limited. Previously, we reported that glucosylceramide enhances the anticoagulant actions of activated protein C with physiologic significance [20] and that sphingosine (2-amino-4-octadecene-1,3-diol) and its analogs down-regulate IIa generation by the II-ase complex [21]. Sulfatide is another sphingolipid that can trigger contact activation, at least in part, by binding factor XII with high affinity [22,23]. Lysosulfatides (lyso-SF) (Fig 1), the deacylated form of sulfatides, are plasma lipids present in cells and in High Density Lipoprotein (HDL) particles [24–26] and lyso-SF is reported to regulate extracellular signaling possible via S1P3 receptor [24,27–29]. However, the influence of lyso-SF on the activities of the plasma blood coagulation system is not known.

In this study, we report that lyso-SF inhibited IIa generation by the II-ase complex. Lyso-SF was bound by both fXa and Gla domainless (gd)-fXa, and it inhibited IIa production both in the presence and absence of fVa. The free amine was required for the antithrombotic activity of lyso-SF since N-acetyl sulfatide did not inhibit II-ase activity. In summary, these findings suggest that soluble lyso-SF is an anticoagulant lipid that binds fXa outside its Gla domain. Lyso-SF and sphingosine and their analogs may point towards a new class of antithrombotic lyso-lipids that could be attractive pharmacologic targets for anticoagulant therapy.

## Materials and Methods

### Materials

FVa, fXa, des Gla (gd)-fXa, II and biotin-GluGlyArg chloromethylketone (BEGR-ck), were obtained from Haematologic Technologies Inc., Essex Junction, VT. For some assays II, was obtained from Enzyme Research Laboratories, South Bend, IN. Lyso-sulfatide (lyso-SF), N-acetyl-sulfatide, psychosine and glucopsychosine were purchased from Matreya LLC., Pleasant Gap, PA. Chromogenic substrate PefaChrome TH was purchased from Pentapharm Ltd., Basel, Switzerland and S2765 from DiaPharma, West Chester, OH. Lysosphingomyelin, L-α-phosphatidylcholine (chicken egg) and L-α-phosphatidylserine (porcine brain) were obtained from Avanti Polar Lipids, Alabaster AL. L-3-Phosphatidylcholine-1,2-di [1-^14^C]oleoyl PC was purchased from Amersham Biosciences. For some studies, L-α-phosphatidylserine (bovine brain) and L-α-phosphatidylcholine (bovine brain) were purchased from Sigma-Aldrich Co. PL vesicles containing phosphatidylcholine (PC) and phosphatidylserine (PS) in the molar ratio 9:1 were prepared by sonication and centrifugation as described earlier [30]. Normal human plasma was purchased from George King Biomedical Inc., Overland Park, KA. For the SPR experiments, streptavidin (SA) sensor chips were obtained from GE Healthcare, Piscataway, NJ. Dansyl-GluGylArg chloromethylketone (DEGR-ck) was obtained from EMD Biosciences, San Diego, CA. All other chemicals and reagents were of the highest purity available.

### Handling and Storage of Lyso-SF and Its Analogs

Lyso-SF and its analogs were solubilized from lyophilized powder state immediately prior to use. Typically, lyophilized powder of lyso-SF or its analogs were dissolved in 50 mM Hepes (pH 7.4), 150 mM NaCl, 0.5% bovine serum albumin containing buffer (abbreviated HBS-BSA buffer) to a final concentration of 1 mg/mL. Lyso-SF solutions were stored at 4°C and solutions were discarded after two days of storage to avoid artifacts due to oxidation of the lipids.

### Critical Micellar Concentration (CMC) Determination

The CMC of lysoSF was determined using diphenyl hexatriene (DPH) at room temperature as previously described with some modification (21). Briefly, 50 μL of DPH dissolved in dimethyl formamide (1 in 400 dilution in 20 mM Tris, 100 mM NaCl, 0.5% BSA, pH 7.4 (TBSA) or dimethyl formamide (1 in 400 dilution in TBSA) were added to 60 μL of various concentrations of lyso-SF and then incubated for 45 min at room temperature. Then, DHP fluorescence was monitored at 365 nm excitation and 460 nm emission, respectively. The net initial emission intensity, termed F0, was obtained by subtracting the initial intensity of dimethyl formamide from the initial intensity of DPH. Relative fluorescence intensity (F/F0) was calculated for expressing the change of fluorescence intensity. F was the net emission intensity of a sample at a given point in the lipid titration. When lyso-SF was added at room temperature to a buffer solution containing DHP (final 25 μM) in HBS containing 0.5% BSA, a shallow, concentration-dependent, linear increase in DPH fluorescence was observed up to 300 μM; and there was no inflection point or break in the linear slope. Since this method is broadly used to detect CMC values that are seen as an inflection point with a sharp transition to a steeper slope in this plot, the CMC of lyso-SF under these buffer and temperature conditions was above 300 μM (data not shown).

### fXa-1-Stage Assays

The anticoagulant properties of lyso-SF and its analogs were determined by fXa-initiated clotting time using normal pooled plasma. Briefly, normal human plasma (NHP) (7.5 μl) of was mixed with varying doses of lyso-SF or its analogs in HBS-BSA buffer (40 μL) and fibrinogen (10 μL, 0.6 mg/ml final) and incubated for 3 min at 37°C. After addition of fXa (50 μl, 1.5 nM, final) containing 15 mM CaCl_2_, clotting times were recorded using an Amelung KC4 micro coagulometer (Sigma Diagnostics, St Louis, MO). Clotting times were converted to residual fXa activity using a fXa standard curve in order to calculate the IC_50_ of lyso-SF inhibitory effect on fXa in plasma.

### IIa-Time Clotting Assays

NHP (7.5 μL) was mixed with lyso-SF (20 μL, varying concentrations), fibrinogen (50 μL, 0.6 mg/mL final) and 50 μL of HBS-BSA buffer and incubated at 37°C for 3 min. Clotting was initiated by the addition of IIa (50 μL, 0.43 units/mL, final) in buffer containing 30 mM CaCl_2_.

### Amidolytic Activity Assays

FXa or IIa (5 nM, final) in the presence or absence of PC/PS vesicles (25 μM final) were incubated with various concentrations of lyso-SF (ranging between 0–80 μM) before the addition of 0.6 mM of either S2765 or Pefachrome TH substrates, respectively. Generation of *p*-nitroaniline was measured at 405 nm using an OPTIMAX plate reader (Molecular Devices, Sunnyvale, CA).

### II-ase Assays

Effects of lyso-SF and its analogs on II activation by II-ase were measured in a two-stage assay. The first step involved the generation of IIa, which was subsequently measured in the second step using the IIa chromogenic substrate PefaChrome TH. Since both meizo-thrombin and α-IIa exhibit similar amidolytic activity, we herein describe II activation without distinguishing meizo-thrombin from α-IIa. Amounts of fXa, PL, and II present in the assay were such that the rate of IIa generation was linearly proportional to the amount of fVa present in the reaction mixture. In some experiments, varying concentrations of lyso-SF were incubated with fVa (0.16 nM, final) in HBS-BSA plus 5 mM CaCl_2_, 0.1 mM MnCl_2_ (abbreviated as Low Salt Binding Buffer, LSBB), II (0.6 μM final) and PC/PS vesicles (25 μM final) in LSBB. IIa generation was initiated by the addition of fXa (0.08 nM final). After the reaction was allowed to proceed for 3 min, IIa generation was stopped by the addition of 50 mM Tris (pH 7.4), 100 mM NaCl, 50 mM EDTA, 0.02% NaN_3_, 0.05% BSA, and IIa formation was quantified by measuring the rate of substrate (PefaChrome-TH, 0.4 mM final) hydrolysis at 405 nm.

In the absence of PL, II activation was also determined using fXa alone or fXa plus fVa. II (0.75 μM final) was mixed with various concentrations of lyso-SF at room temperature and then incubated with fXa (0.75 nM, final) in the presence or absence of fVa (15.4 nM, final) for 5 min and 120 min, respectively. In experiments where either gd-fXa was used in place or fXa, the same general protocols were followed except that the incubation times were adjusted for the generation of detectable amounts of IIa. After detectable IIa was generated, the II-ase reaction was quenched by EDTA, and the rate of IIa formation over time was determined following the same protocol as given above.

### Labeling of fXa and GD-fXa for Biophysical Characterizations

FXa was active site-specifically labeled with either biotin or a dansyl probe for the biophysical studies. Briefly, fXa (2.9 μM) in 50 mM Hepes (pH 7.4), 150 mM NaCl, 5 mM CaCl_2_ was incubated with a five-fold molar excess of either biotin-GluGlyArg(BEGR-)chloromethylketone (ck) or dansyl-GluGlyArg-ck (DEGR-ck) to generate inactive (i) BEGR-fXa_i_ or DEGR-fXa_i_, respectively. The excess inhibitor was dialyzed away from the product after > 99% of the fXa was inhibited as estimated by chromogenic assays using S2222. The labeled proteins were quantified for dye-to-protein ratio using a molecular weight of 46,000 Da and a ε^1%^_1 cm_ at 280 nm of 11.6 for human fXa and assuming an ε_334_ of 4,600 M^-1^cm^-1^ for DEGR-ck [31]. A dye-to-protein ratio of 1.1:1 was obtained for these preparations indicating that each fXa molecule contains 1 dye molecule on average.

### Spectral Measurements

Steady state fluorescence intensity measurements were made using a SLM AB2 spectrofluorometer (SLM-Aminco, Rochester, NY) as described [32]. Dansyl emission was detected using excitation at 340 nm and emission at 530 nm. The bandpass width was 8 nm on the excitation beam and 16 nm on the emission beam for experiments involving DEGR-fXa_i_. Steady state anisotropy measurements were performed as described [33]. All experiments were performed using 5 x 5 mm quartz cuvettes. Samples were mixed and adsorption of proteins to cuvette walls were minimized as described [34,35].

### Coupling of Biotin-Labeled Proteins to Sensor Chip

Coupling of BEGR-fXa and BEGR-gd-fXa to the gold surface of the sensor chip was achieved by flowing 100 μg/ml of biotin-labeled proteins in 10 mM Hepes buffer, 300 mM NaCl, 5 mM CaCl_2_ (pH 7.4), over a Biacore SA sensor chip (GE Healthcare, Piscataway, NJ). The amount of protein immobilized was determined after washing to be ~500 units. Coupling was carried out at 300 mM NaCl in order to collapse the dextran matrix and thus minimize artifacts that might occur during the experiments. A control surface was prepared by flowing free biotin (0.003 mg/mL) over a second channel of the SA sensor chip and data from this ‘blank’ channel was subtracted from the sample data.

### Surface Plasmon Resonance (SPR) Studies

SPR Sensorgrams were collected for different lyso-SF concentrations and a single PC/PS vesicle concentration that flowed over a sensor chip containing SA-biotin-fXai or SA-biotin-gd-fXai or SA-biotin (present in the control flow cell) in 10 mM Hepes buffer, 150 mM NaCl, 5 mM CaCl_2_ (pH 7.4). Preliminary experiments revealed a flow rate dependence on the dissociation rate constant (k_d_) in which flow rates less than 50 μl/min showed increasing k_d_ with increasing flow rates. All experiments were therefore carried out at the maximum flow rate of 100 μl/minute and at a sampling rate of 1 Hz on a Biacore 3000. Surface regeneration was carried out using 2.0 M NaCl at 100 μl/minute flow rate for 2 min, following which 50 mM NaOH was injected at 50 μl/minute flow rate to prepare the surface for the next run. Rate constants for association (k_a_) and dissociation (k_d_) were obtained by globally fitting the data from five to six injections of lyso-SF by using the BIAevaluation software version 3.2, using the simple Langmuir binding model. Statistical analysis of the curve fits for both dissociation and association phases of the sensorgrams show low χ^2^ values (0.3–2.2) and low residuals.

## Results

### Effect of Lyso-SF on Plasma Clotting Assays

**Fig 2:**
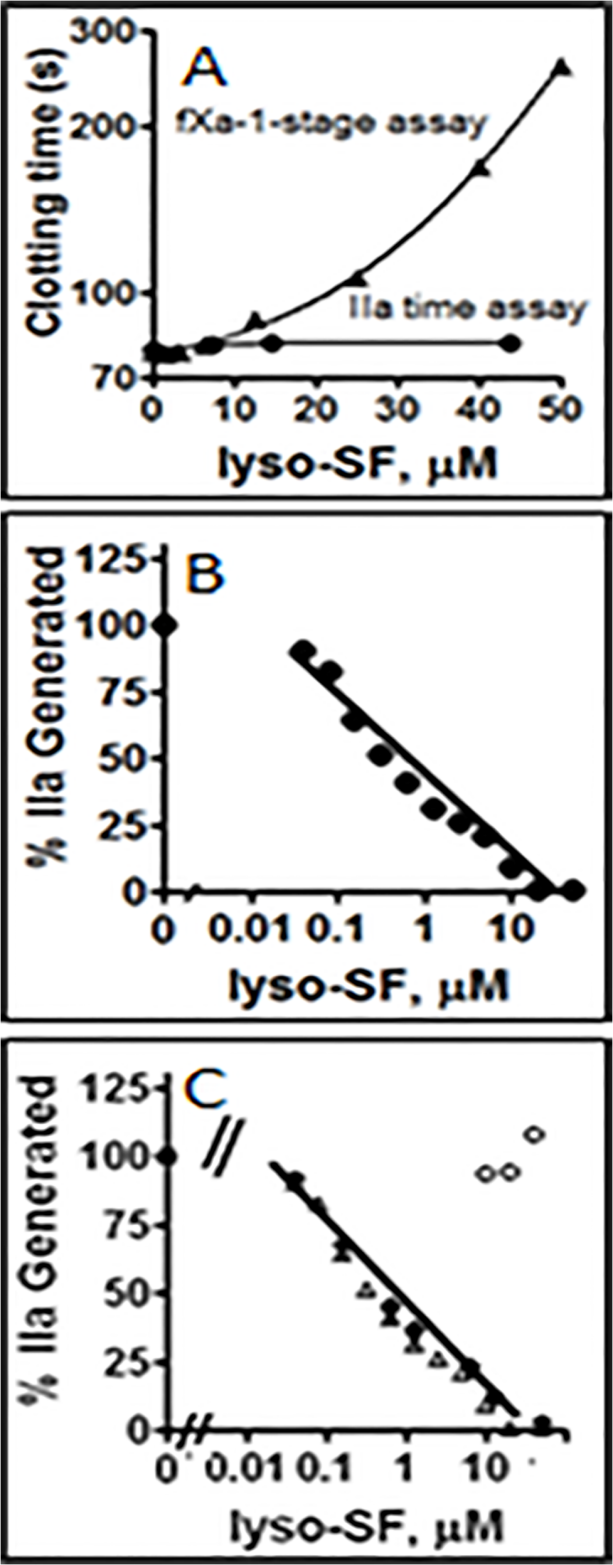
Inhibitory activity of lyso-SF. **(A)** Effects of Lyso-SF in plasma clotting assays: The effects of lyso-SF on fXa-initiated clotting assays (closed circles) or on IIa-initiated clotting assays (open circles). **(B)** Effect of Lyso-SF in II-ase assays: The effects of lyso-SF on IIa generation by the II-ase complex were tested in a purified system (see Experimental Procedures). **(C)** Phospholipid dependence of Lyso-SF inhibition of II-ase: The effects of lyso-SF on IIa generation by the II-ase complex were tested at three different PC/PS vesicle concentrations, no PC/PS vesicles (open circles), 6 μM PC/PS (closed inverted triangles), 15 μM PC/PS (open triangles) and 30 μM PC/PS (closed diamonds).

To test the effect of lyso-SF on plasma clotting reactions, fXa-induced clotting assays and IIa-induced clotting assays were performed in the presence of varying concentrations of lyso-SF. Lyso-SF inhibited fXa-induced coagulation, reflected in clotting time prolongation, in a concentration-dependent manner (Fig 2A). IIa-induced clotting of plasma (i.e., the IIa time assay) was not affected by lyso-SF at the concentrations employed for the fXa-induced clotting assays (Fig 2A) indicating that lyso-SF inhibited prothrombin activation but not the clotting activity of IIa on fibrinogen. The lyso-SF inhibitory effects on fXa had an IC_50_ of ~ 26 μM in the plasma milieu on the fXa-induced clotting assay.

### Effect of Lyso-SF on II-ase Activity

When lyso-SF was tested in purified systems for its ability to inhibit the activation of II by fXa/fVa in the presence of PL, it dose-dependently inhibited II activation by II-ase with an IC_50_ of ~ 0.6 μM (Fig 2B). This lyso-SF dependent inhibition of II-ase activity was not due to the inhibition of IIa enzymatic activity in the second step of the two step assay since lyso-SF did not inhibit IIa amidolytic activity against the small peptide chromogenic substrate, PefaChrome TH (data not shown). Furthermore, the inhibitory effect of lyso-SF was also not due to a decreased capacity of fXa active site to cleave substrates in the presence of lyso-SF since the amidolytic activity of fXa towards its small peptide chromogenic substrate, S2765, was unaltered by lyso-SF (data not shown). These data show that lyso-SF had a direct inhibitory effect on IIa generation by the II-ase complex.

### Phospholipid-Dependence of Lyso-SF Inhibition of II-ase

To test if lyso-SF inhibits II-ase by competing with PC/PS vesicles for binding components of the II-ase complex, lyso-SF inhibition of II-ase was tested at four different total PL concentrations, namely 0, 6, 15, and 30 μM PL, respectively. When PL was present, Lyso-SF inhibition of II-ase was similar at 6 μM (Fig 2C), 15 μM (data not shown) and 30 μM PL with an IC_50_ ~ 0.6 μM, suggesting that the mechanism of lyso-SF inhibition of II-ase was something other than simply competition of lyso-SF with PL for protein binding (Fig 2C). In reactions lacking PL, II activation by fXa/fVa was not inhibited by lyso-SF (Fig 2C, open circles). These data show that PL was required for lyso-SF-dependent inhibition of II-ase but that lyso-SF did not compete for PL-clotting factor interactions.

### FVa-Dependence of Lyso-SF Inhibition of II Activation

II activation occurs primarily via a meizo-thrombin intermediate in the presence of fVa whereas it proceeds via a prethrombin-2 intermediate in the absence of fVa [1,33,36]. Earlier we showed that sphingosine (Fig 1), which lacks the sulfogalactosyl moiety of lyso-SF, requires fVa for its inhibition of II activation [21]. However, lyso-SF inhibited II activation by fXa in the absence of fVa (S1 Fig), although much less potently. In the absence versus presence of fVa, the IC_50_ values were 35 μM (S1 Fig) versus 0.6 μM (Fig 2B and 2C). Therefore, fVa greatly augments the potency for lyso-SF inhibition. Moreover, lyso-SF did not alter significantly the K_m_ for II which randomly ranged from 0.13 to 0.21 μM for the curves in S1 Fig

### Gla Domain Requirement for Lyso-SF Inhibition of fXa in II-ase

Since the classical paradigm posits that lipid binding sites are localized in the amino terminal Gla-domain of clotting factors [1], we tested if lyso-SF would inhibit II activation when Gla-domainless (gd)-fXa was used to activate II in the presence of PL. Lyso-SF inhibited II activation by gd-fXa•fVa•PC/PS (S2 Fig). Thus, the Gla-domain of fXa was not absolutely required for the inhibitory effects of lyso-SF. However, when II activation was determined using gd-fXa/PL in the absence of fVa, lyso-SF was not inhibitory (S2 Fig), showing that at least either the Gla domain or fVa was required. The IC_50_ in the absence of the Gla domain was 12 μM for gd-fXa (S2 Fig) versus 0.6 μM for intact fXa (Fig 2B and 2C). Thus, the Gla domain, like fVa, augments the potency for lyso-SF inhibition of II-ase activity.

### Inhibition of II Activation by Analogs of Lyso-SF

**Fig 3:**
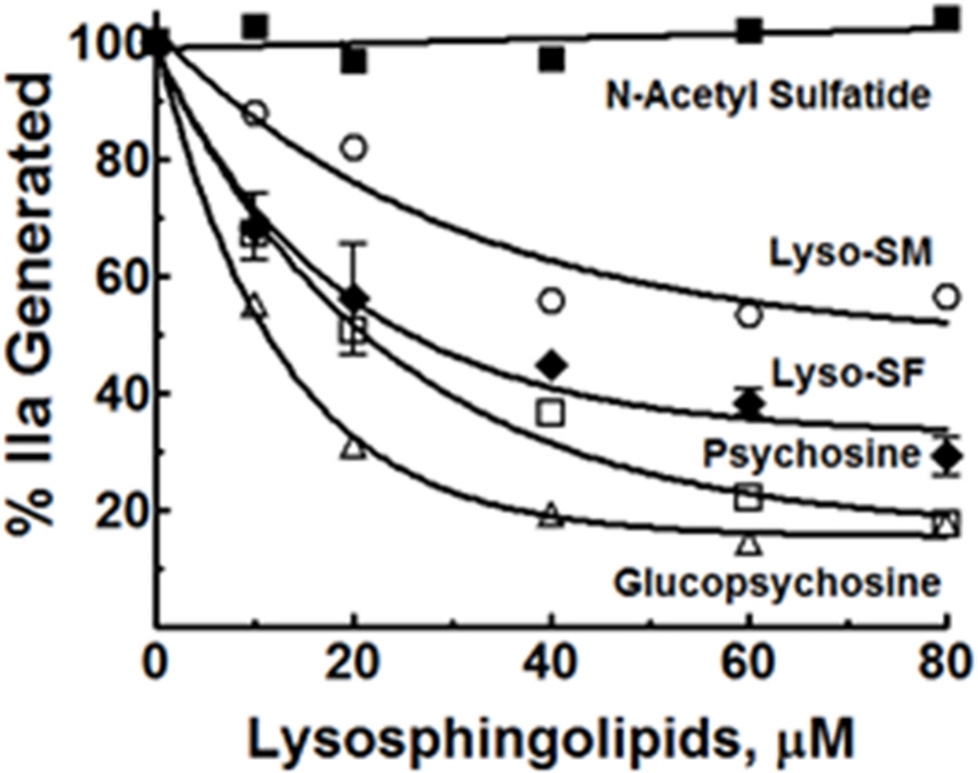
Effect of different analogs of Lyso-SF on II activation by gd-fXa/fVa/PL. The effects of lyso-SF (closed diamonds), psychosine (open squares), glucopsychosine (open triangles), lyso-sphingomyelin (open circles), and N-acetyl sulfatide (closed squares) on the activation of II by gd-fXa/fVa/PL are shown.

Lyso-SF is zwitter ionic at physiologic pH of 7.4, with an acidic sulfate (pKa ~ 1.9) moiety and a basic amino group (pKa ~ 10). Psychosine (*desulfato* lyso-SF, Fig 1) lacks the acidic sulfate ester moiety and is therefore positively charged at physiologic pH. The requirement for the sulfate ester moiety in lyso-SF for the inhibition of II activation by gd-fXa/fVa/PL was tested by using psychosine instead of lyso-SF. gd-fXa was used to maintain focus on the effects of sphingolipids on fXa activity due to the lipid’s binding outside the Gla domain (see below). Psychosine inhibited II activation by gd-fXa/fVa/PL as potently as did lyso-SF (Fig 3). This suggests that the sulfate ester in lyso-SF is not necessary for its inhibition of IIa generation by gd-fXa.

To assess the importance of the sugar group, gluco-psychosine (Fig 1) was used instead of lyso-SF (which contains the diastereomer galactose for sugar). Gluco-psychosine inhibited II activation by gd-fXa/fVa/PL slightly more efficiently than lyso-SF (Fig 3) suggesting that the orientation of the alcohol functionality on carbon 4 of the aldohexose is not important for the expression of the anticoagulant activity of lyso-SF. To assess further the importance of the aldohexose ring as a whole in this reaction, lyso-sphingomyelin (lyso-SM, Fig 1) was used in place of lyso-SF. Lyso-SM contains a phosphorylcholine moiety attached to carbon 1 of sphingosine imparting to it an overall positive charge at physiologic pH of 7.4. Lyso-SM inhibited II activation by gd-fXa/fVa/PL suggesting that the aldohexose ring, as such, was not essential for the inhibitory effects of lyso-SF (Fig 3, circles).

Sphingosine requires a free amine on carbon 2 for its anticoagulant effects [21]. Next we tested to see if this amino group was necessary for the inhibitory effects of lyso-SF. N-acetyl-sulfatide, lacking the amino group (Fig 1), did not inhibit II-ase, showing that the free amino group at carbon 2 on lyso-SF was absolutely required for its inhibitory activity (Fig 3, filled squares).

### Lyso-SF Binding to BEGR-fXa_i_ and BEGR-gd-fXa_i_

**Fig 4:**
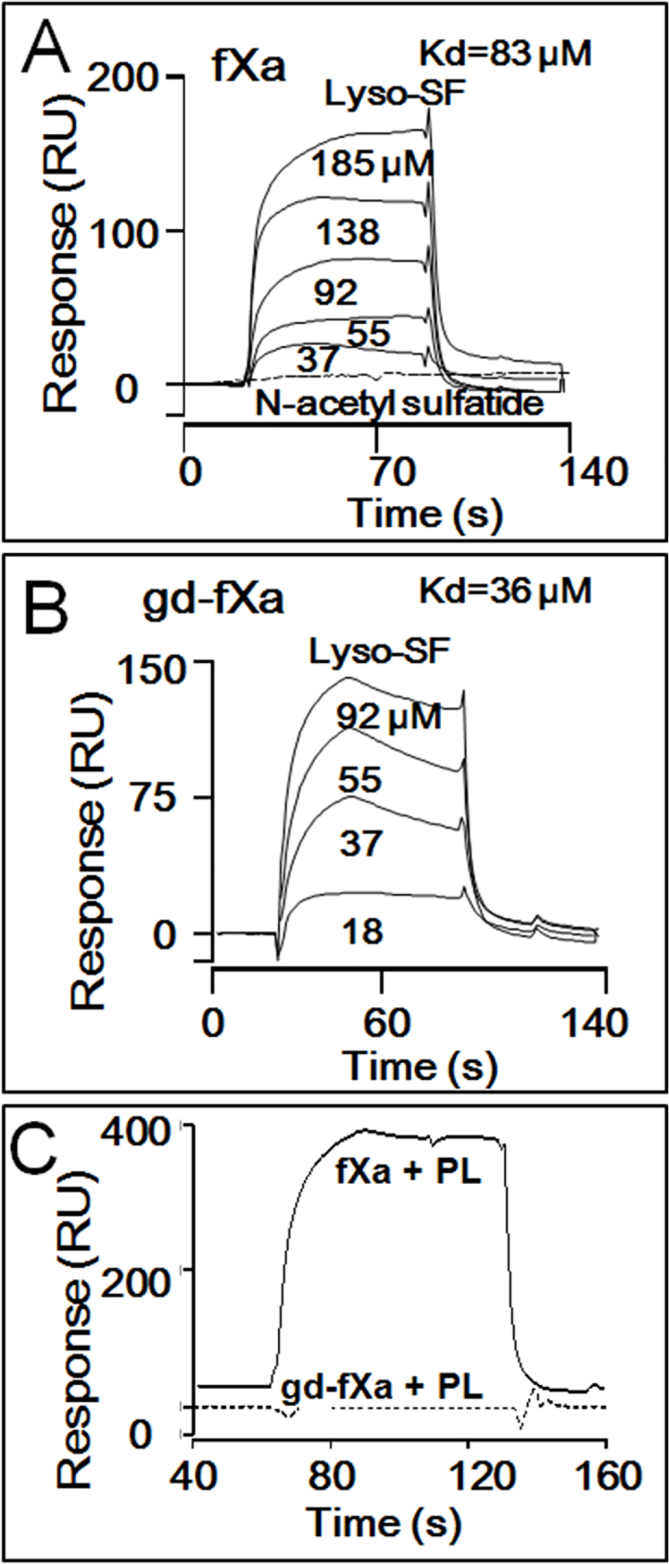
Binding of Lyso-SF to fXa and Gd-fXa using SPR. Surface Plasmon Resonance (SPR) was used to monitor binding of lyso-SF to BEGR-fXa_i_ and BEGR-gd-fXa_i_. **(A)** Sensorgram depicting the dose-dependent binding of lyso-SF (from top to bottom; 185, 138, 92, 55, 37μM) to BEGR-fXa_i_. Lyso-SF (62.5 μM) did not exhibit any binding to N-acetylsulfatide (dotted line). **(B)** Sensorgram depicting the dose-dependent binding of lyso-SF (from top to bottom; 92, 55, 37, 18 μM) to BEGR-gd-fXa_i_. **(C)** PC/PS vesicles exhibited binding to BEGR-fXa_i_ but not to BEGR-gd-fXa_i_.

Surface Plasmon Resonance (SPR) analysis was used to assess and compare binding of biotinylated, inactivated BEGR-fXa_i_ (Fig 4A) and BEGR-gd-fXa_i_ (Fig 4B) to lyso-SF. Both proteins exhibited concentration-dependent binding of lyso-SF. Analysis of the sensorgrams indicated that BEGR-fXa_i_ and BEGR-gd-fXa_i_ bound lyso-SF with apparent affinities of 83 μM and 36 μM, respectively. Importantly, N-acetyl sulfatide at 125 μM that was functionally inactive as an anticoagulant lipid, did not show any binding to BEGR-gd-fXa_i_ (Fig 4A). In control experiments, when the binding of lyso-SF to two vitamin K-dependent homologs of fXa was studied, BEGR-fVIIa_i_ and BEGR-fIXa_i_ showed no detectable binding of lyso-SF (data not shown). Thus, the binding of lyso-SF to fXa and gd-fXa was quite specific. In other controls, PL vesicles exhibited binding to BEGR-fXa_i_, but not to BEGR-gd-fXa_i_ (data not shown), consistent with the classical paradigm that the amino terminal Gla-domain of fXa is responsible for fXa binding to PL vesicles. These binding data are consistent with the hypothesis that a direct interaction between gd-fXa and lyso-SF is critical for the inhibitory effects of the lyso-SF.

### Lyso-SF Binding to DEGR-fXa_i_ in the Presence and Absence of PC/PS

**Fig 5:**
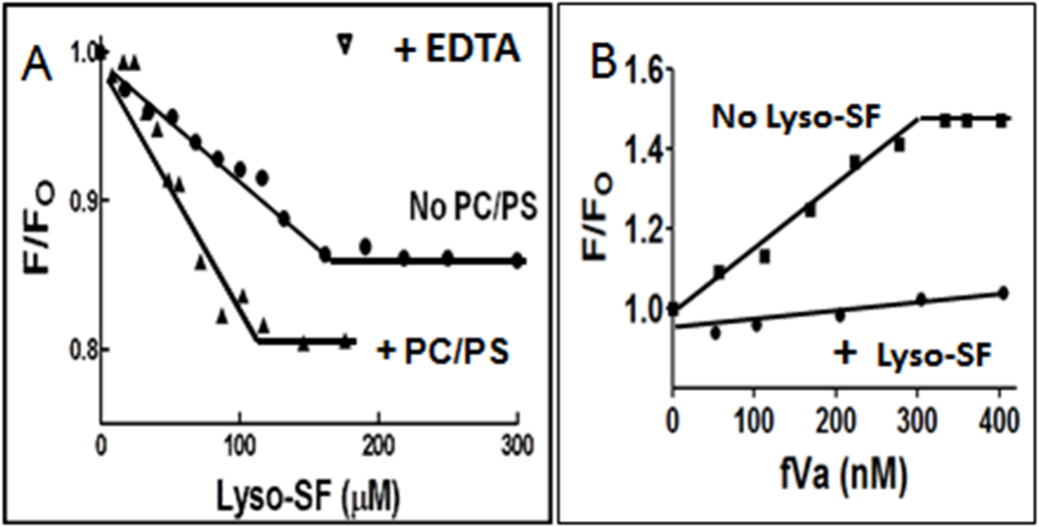
Fluorescence spectroscopy binding studies. **(A)** Binding of Lyso-SF to fXa in the presence or absence of PL Vesicles. The binding of fXa to lyso-SF was monitored by fluorescence spectroscopy. Samples containing DEGR-fXa_i_ (initially 200 nM) in 50 mM Hepes (pH 7.4), 150 mM NaCl, 5 mM CaCl_2_ either in the presence (closed triangles) or absence (closed circles) of PC/PS vesicles were titrated with lyso-SF and the net fluorescence intensity recorded. At the end of the titration, EDTA (10 mM) was added to reverse the fluorescence change. F was the fluorescence intensity at any given point in the titration and F_o_ was the initial fluorescence intensity, before the addition of lyso-SF. **(B)** Binding of FVa to DEGR-fXa/PL in the presence or absence of Lyso-SF. DEGR-fXa_i_ (initially 200 nM) in 50 mM Hepes (pH 7.4), 150 mM NaCl, 5 mM CaCl_2_ was titrated with 25 μM PC/PS vesicles prior to the addition of 100 μM lyso-SF (closed circles) or buffer (closed squares). Subsequently the complex was titrated with fVa and the net fluorescence intensity of DEGR-fXa_i_ recorded at λ_ex_ = 340 nm and λ_em_ = 530 nm, respectively. F was the fluorescence intensity at any given point in the titration and F_o_ was the initial fluorescence intensity before the addition of lyso-SF.

Due to the PC/PS requirement for the lyso-SF inhibition of II-ase activity, the binding of lyso-SF to DEGR-fXa_i_ was monitored in the presence or absence of PC/PS vesicles using fluorescence spectroscopy. When lyso-SF was added to DEGR-fXa_i_ in the absence and presence of PC/PS vesicles, the net dilution-corrected steady state emission intensity of the dansyl moiety in DEGR-fXa_i_ decreased and the change reached a plateau (Fig 5A). The net change in fluorescence was greater in the presence than absence of PC/PS, and the lyso-SF concentration for half-maximal change (EC_50_) was 50 μM in the presence of PC/PS versus 80 μM in the absence of PC/PS vesicles (Fig 5A). Thus, when DEGR-fXa_i_ was bound to PC/PS vesicles, it actually bound lyso-SF with a greater affinity and it induced a more robust change in dansyl fluorescence. At the end of the titrations, when a molar excess of EDTA was added to chelate the calcium ions in the solution (Fig 5A, inverse triangle), the fluorescence changes observed due to the addition of lyso-SF and PC/PS to DEGR-fXa_i_ were completely reversed, showing that the conformational changes monitored by dansyl fluorescence were completely reversible.

### Binding of FVa to DEGR-fXa_i_ in the Presence and Absence of Lyso-SF

To test if lyso-SF interfered with fXa-fVa interactions, the binding of fVa to the DEGR-fXa_i_•PC/PS complex was monitored in the presence or absence of lyso-SF. For these studies, two titrations were done in parallel. Initially, DEGR-fXa_i_ was titrated with PC/PS vesicles, to elicit a small 1% decrease in steady state fluorescence intensity of the dansyl probe in DEGR-fXa_i_. Subsequently, either buffer or 100 μM lyso-SF was added to the DEGR-fXa_i_•PC/PS complex mixture. Then, fVa was added to the samples containing or lacking lyso-SF (Fig 5B). When fVa was added to the control samples lacking lyso-SF, the dansyl emission intensity rose sharply by ~ 48% and reached a plateau at > 300 nM fVa, reflecting fVa binding to the DEGR-fXa_i_•PC/PS complex. However, when fVa was titrated into samples containing lyso-SF, the change in fluorescence signal of the dansyl moiety in DEGR-fXa_i_•PC/PS complex was significantly weaker compared with the fluorescence signal in the absence of lyso-SF. Thus, it appeared that lyso-SF prevented the fVa-induced conformational changes in fXa and/or inhibited fVa binding to fXa (Fig 5B), thereby preventing fVa-induced conformational changes in fXa.

## Discussion

Although phospholipids are well-recognized for their effects on coagulation reactions, little is generally known about the effects of sphingolipids on clotting pathways. Negatively-charged sulfatides can potently initiate the intrinsic pathway of coagulation system by binding and auto-activating fXII [37]. Here we report the discovery that lyso-SF and some of its analogs are anticoagulant single-chain lipids in fXa-induced clotting assays in plasma. Lyso-SF prolonged fXa-initiated clotting times in fXa-1-stage assays without inhibiting the enzymatic activity of thrombin, indicating that lyso-SF inhibits thrombin generation by the II-ase complex rather than fibrin formation by thrombin. Studies showed that lyso-SF was anticoagulant in assays of thrombin generation using purified clotting factors and PL in II-ase assays. Earlier we reported that sphingosine (2-amino-4-octadecene-1,3-diol) inhibits IIa generation by the II-ase complex by binding to the Gla-domain of fXa and disrupting the fXa-fVa interaction [21]. However, mechanisms for lyso-SF anticoagulant activity are notably distinct from those for sphingosine. The potential physiologic significance of sphingolipid inhibition of coagulation is unclear. Nofer et al [24,38] using purified HDL fraction from human plasma determined that the concentration of lyso-SF in the HDL lipoprotein fraction was in the 12–17 μM range using time-of-flight ion mass spectroscopy (TOF-SIMS). However, recent LC/MS/MS methods using plasma instead of HDL as a source of lyso-SF found only pM concentrations of free lyso-SF [39]. This disparity on the results could be explained by the differences on lipid extraction methodology of the samples or the instruments used to analyze the sphingolipids. This findings are comparable to the 0.1–1.2 μM concentrations of sphingosine 1-phosphate, another active sphingolipid that circulates in plasma mostly bound to the HDL lipoprotein fraction [40]. The concentration of sphingosine 1-phosphate in serum is even higher because it is stored at high concentrations and is thought to be released from platelets and other blood cells [41]. More work needs to be done to establish the normal range of concentrations of free and lipoprotein-bound lyso-SF in plasma and serum. While the baseline plasma concentrations of lyso-SF are important to help discuss physiologic significance for the anticoagulant effect of lyso-SF, the local concentration of lyso-SF could increase dramatically at the site of injury by the release of intracellular lyso-SF deposits. Other factors could also influence the local concentration of lyso-SF, like upregulation of enzymatic activities such as the sulfatide N-deacylase responsible for the degradation of sulfatide into lyso-SF and alterations of the activity of arylsulfatase A responsible for the cellular catabolism of lyso-SF [42]. Nonetheless, significant insights into structure-activity relationships for the lipid and for lipid-protein interactions are defined here. The EC_50_ and IC_50_ parameters obtained in the plasma assays, purified II-ase assays, and fXa binding experiments, generally agreed on establishing a low to mid μM range for lyso-SF to exert its anticoagulant effects.

Lyso-SF is composed of a sphingosine-like moiety with a primary amine on the second carbon and a hydroxyl group on carbon three. Studies of the anticoagulant activity of lyso-SF analogs, including N-acetyl sulfatide, showed that the free amino group on carbon 2 was absolutely required for its II-ase inhibitory activity.

Although the mechanisms for II-ase inhibition by lyso-SF are not completely clear, a number of insights were obtained about whether lipid-lipid or lipid-protein interactions were important. Lyso-SF did require the presence of PL to inhibit fXa activity in the absence of fVa. To test if the inhibitory activities of lyso-SF were due to its disruption of PL vesicles or due to competition with PL vesicles for binding protein components of the II-ase complex, lyso-SF inhibition of II-ase was studied in the absence of PL and in the presence of multiple concentrations of PL. Lyso-SF inhibited II-ase activity similarly at both low and high concentrations of PL, suggesting that lyso-SF does not disrupt PL vesicle structure or compete with PC/PS vesicles for binding fXa/fVa. Furthermore, PL vesicles containing 20% PS optimally decrease the K_m_ for II in II-ase assays [4] and II activation by fXa/PCPS was inhibited by lyso-SF without changing this Km. This also suggests that the anticoagulant effect of lyso-SF is not due to the PL vesicle blockade. Notably, lyso-SF is anticoagulantly active in plasma with an ~ IC_50_ of 26 μM obtained on plasma clotting assays. However, plasma contains very high levels of lipids and lipoproteins where the concentration of lyso-SF is too low to compete with other lipid coagulation surfaces. Moreover, the EC_50_ obtained for lyso-SF anticoagulant activity in purified systems in the presence and absence of PL vesicles was similar (50 vs 80 μM) which argue against a role for PL vesicles on the mechanism of action of lyso-SF. These multiple considerations make it quite unlikely that Lyso-SF interferes with PL-clotting factor interactions.

To gain mechanistic insights for direct interactions of lyso-SF with clotting factors, SPR and fluorescence spectroscopy were employed. Lyso-SF bound specifically to both fXa and gd-fXa in SPR assays whereas no binding was observed in controls using two other vitamin K-dependent factors, fVIIa and fIXa. The lyso-SF binding affinities obtained for fXa and gd-fXa were similar suggesting that lyso-SF has interaction site(s) outside of the gla-domain of fXa. Importantly, functionally inactive N-acetyl sulfatide did not bind gd-fXa, suggesting that the binding of lyso-SF to regions other than the Gla domain of fXa may be critical for the inhibitory effects of lyso-SF. To clarify if fVa was required for the lyso-SF-dependent inhibition of II-ase, assays were performed in the absence of fVa. Lyso-SF inhibited II activation by fXa/PL even in the absence of fVa, indicating that this lipid-fVa interaction was not required for lyso-SF anticoagulant activity. Nonetheless, when fVa is present, it remains possible that this lysolipid-fVa interaction might favorably enhance the inhibition of II-ase activity.

Previous work indicates that the conformation of the protease domain of fXa is altered from a less active state to a more active state by binding to PL membranes [14,15,43] or to fVa [44–47]. Lyso-SF inhibited II activation by gd-fXa/fVa/PL and activation by fXa/fVa/PL. However, lyso-SF did not inhibit the activation of II by gd-fXa/PL. Thus, for lyso-SF inhibition, there was a requirement for fXa to be bound to fVa or to PL in the II-ase complex. These data are consistent with the hypothesis that the activity-enhancing fXa conformational changes induced by either PL or fVa render fXa much more sensitive to inhibition by lyso-SF. Further, since lyso-SF binds gd-fXa, we hypothesize that lyso-SF binds fXa outside its Gla domain to inhibit fXa procoagulant activity whitout inhibiting fXa amidolytic activity. Alternative mechanisms for lyso-SF inhibition are also possible, but we posit the simplest explanation for lyso-SF’s ability to reduce II-ase activity.

In summary, this study provides new biochemical knowledge about the diverse potential effects of sphingolipids, i.e., lyso-SF and several of its analogs, which are potent anticoagulant lipids. This study also extends the still novel, if not controversial, concept that binding of lipids to clotting factors outside Gla domains may contribute to regulate the activity of fXa [16,17,48–53].

## Supporting Information

S1 Fig Lyso-SF inhibition of fXa in the absence of FVa. The effects of lyso-SF on IIa generation by fXa/PL at varying II concentrations were tested (see Experimental Procedures). The inhibitory effects of 0 (open circles), 20 μM (closed squares), 40 μM (closed circles), 60 μM (open squares), and 80 μM (open triangles) lyso-SF are shown.
(TIF)

S2 Fig Influence of the GLA domain on the activity of lyso-SF under different conditions. The effects of lyso-SF on II activation by either gd-fXa/fVa/PL (closed circles) or gd-fXa/PL (open squares) are shown.
(TIF)
