# Supplementary figures and images for: Lyso-Sulfatide Binds Factor Xa and Inhibits Thrombin Generation by the Prothrombinase Complex
Source: PLoS One. 2015 Aug 11;10(8):e0135025. doi: 10.1371/journal.pone.0135025 (PMC4532512; doi:10.1371/journal.pone.0135025)

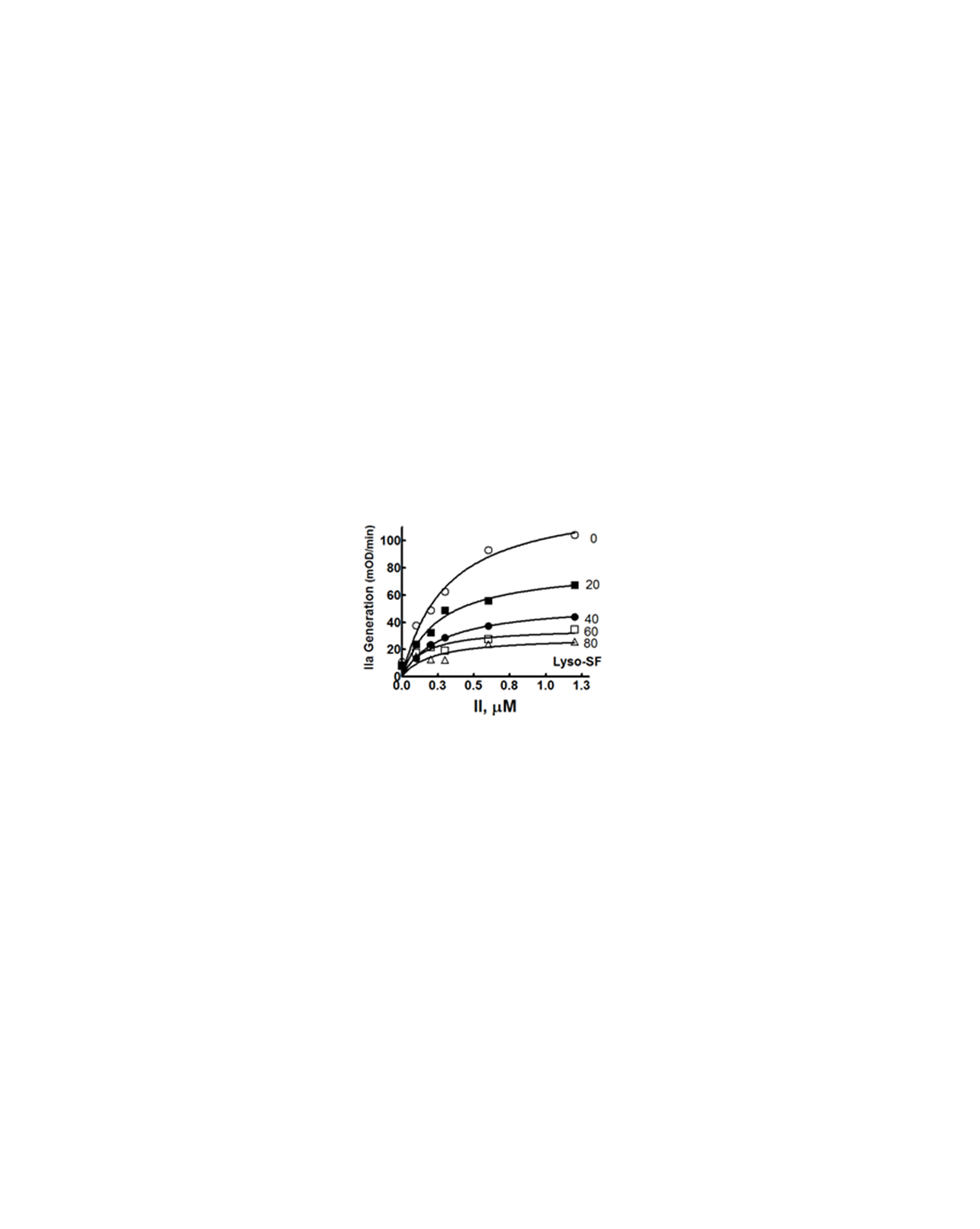

Supplement: S1 Fig — The effects of lyso-SF on IIa generation by fXa/PL at varying II concentrations were tested (see Experimental Procedures). The inhibitory effects of 0 (open circles), 20 μM (closed squares), 40 μM (closed circles), 60 μM (open squares), and 80 μM (open triangles) lyso-SF are shown. (TIF) [file pone.0135025.s001.tif]

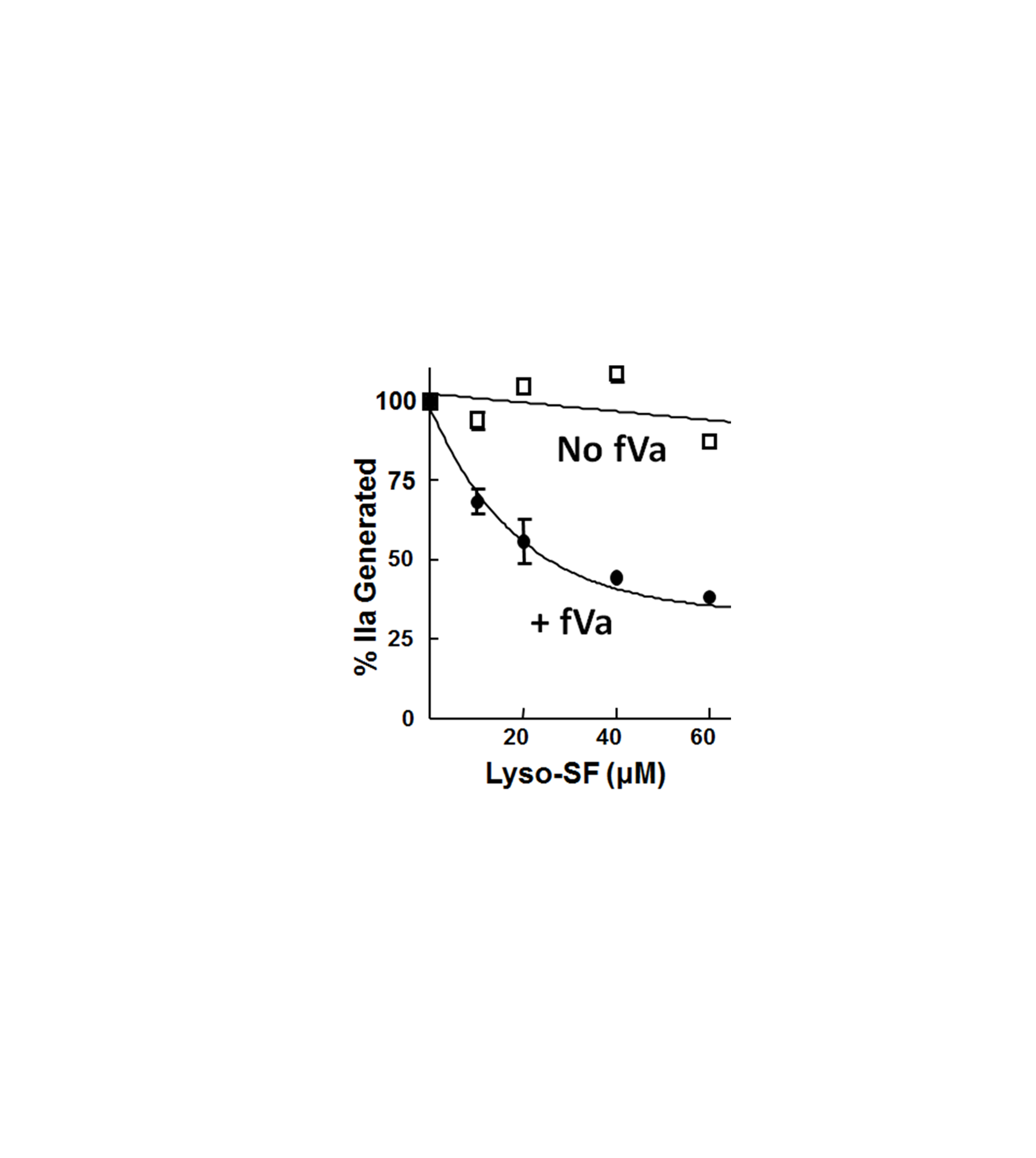

Supplement: S2 Fig — The effects of lyso-SF on II activation by either gd-fXa/fVa/PL (closed circles) or gd-fXa/PL (open squares) are shown. (TIF) [file pone.0135025.s002.tif]
